# A novel risk score system for prognostic evaluation in adenocarcinoma of the oesophagogastric junction: a large population study from the SEER database and our center

**DOI:** 10.1186/s12885-021-08558-1

**Published:** 2021-07-13

**Authors:** Jun Wang, Le Shi, Jing Chen, Beidi Wang, Jia Qi, Guofeng Chen, Muxing Kang, Hang Zhang, Xiaoli Jin, Yi Huang, Zhiqing Zhao, Jianfeng Chen, Bin Song, Jian Chen

**Affiliations:** 1 Department of Gastroenterology Surgery, the Second Affiliated Hospital, Zhejiang University School of Medicine, 88 Jiefang Road, Hangzhou, 310000 China; 2 Department of Gastroenterology Surgery, Shaoxing Shangyu People’s Hospital and Shangyu Hospital of the Second Affiliated Hospital, Zhejiang University School of Medicine, Shaoxing, Zhejiang, 312300 China

**Keywords:** Adenocarcinoma of the oesophagogastric junction, SEER, LASSO method, Prognostic evaluation, External validation

## Abstract

**Background:**

The incidence rate of adenocarcinoma of the oesophagogastric junction (AEG) has significantly increased over the past decades, with a steady increase in morbidity. The aim of this study was to explore a variety of clinical factors to judge the survival outcomes of AEG patients.

**Methods:**

We first obtained the clinical data of AEG patients from the Surveillance, Epidemiology, and End Results Program (SEER) database. Univariate and least absolute shrinkage and selection operator (LASSO) regression models were used to build a risk score system. Patient survival was analysed using the Kaplan-Meier method and the log-rank test. The specificity and sensitivity of the risk score were determined by receiver operating characteristic (ROC) curves. Finally, the internal validation set from the SEER database and external validation sets from our center were used to validate the prognostic power of this model.

**Results:**

We identified a risk score system consisting of six clinical features that can be a good predictor of AEG patient survival. Patients with high risk scores had a significantly worse prognosis than those with low risk scores (log-rank test, *P*-value < 0.0001). Furthermore, the areas under ROC for 3-year and 5-year survival were 0.74 and 0.75, respectively. We also found that the benefits of chemotherapy and radiotherapy were limited to stage III/IV AEG patients in the high-risk group. Using the validation sets, our novel risk score system was proven to have strong prognostic value for AEG patients.

**Conclusions:**

Our results may provide new insights into the prognostic evaluation of AEG.

**Supplementary Information:**

The online version contains supplementary material available at 10.1186/s12885-021-08558-1.

## Background

Adenocarcinoma of the oesophagogastric junction (AEG) refers to a malignancy that crosses the line of the gastroesophageal junction and includes distal oesophageal cancer and proximal gastric cancer. An estimated 604,100 new cases and 544,076 deaths from oesophageal cancer, as well as 1,089,103 new cases and 768,793 deaths from stomach cancer, worldwide were reported in 2020 [1]. The incidence rate of AEG has significantly increased in Western countries over the past two decades [2]. In Asian countries, AEG incidence is reported to be increasing in Malaysia and Japan [3]. In China, an increasing trend of AEG has also been observed over the past 25 years [4]. Over the past three decades, the increase in morbidity has resulted in a steady increase in mortality, from 2 deaths to 15 deaths per 100,000 [5]. The causes of these malignancies include gastroesophageal reflux disease, Barrett’s oesophagus, the use of acid-suppressing drugs, obesity, and smoking. One of the risk factors, Barrett’s adenocarcinoma, has been proven to be a positive clinical subtype of AEG, with the potential risk of spreading through the complex lymphovascular network of the oesophagus [6]. According to the eighth edition of the American Joint Committee on Cancer (AJCC) Cancer Staging Manual, cancers less than 2 cm from the gastric cardia are classified as oesophageal adenocarcinoma (also known as Siewert types I/II), while cancers more than 2 cm from the gastric cardia are classified as gastric cancers (Siewert type III) [7]. However, this manual does not consider the impact of other critical clinical factors, such as age, sex, cancer invasion (T) stage, lymph node metastasis (N) stage, distant metastasis (M) stage or the total number of examined lymph nodes (LNs), which could also be predictive factors that influence AEG patient prognosis [8]. Therefore, we need to consider a variety of factors to judge the outcome of AEG patients.

The Surveillance, Epidemiology, and End Results Program (SEER) database collects data on cancer cases from various locations and sources throughout the United States (https://seer.cancer.gov/data/). The SEER registry contains patient demographic data, the primary tumour site, tumour morphology, the diagnostic stage, and the first course of treatment. Recently, an increasing number of studies on the incidence, diagnosis, treatment, or prognosis of human cancers have been reported based on this important database. For example, for treatment comparisons, these studies focused on hepatocellular carcinoma [9, 10], small cell carcinoma of the oesophagus [11], and oral cavity cancer [12]; and for prognostic evaluation, lymphoma [13], soft tissue sarcomas [14], ovarian cancer [15], testicular choriocarcinoma [16], prostate cancer [17], and colorectal cancer [18]. In lymphoma, Zhong et al. developed a predictive nomogram as a novel risk stratification model for cancer-specific survival in diffuse large B-cell lymphoma patients based on a large cohort from the SEER database [13]. Thus, this inspired us to use clinical cancer data in the SEER database to establish a prognostic evaluation model for AEG patients.

In this study, we obtained clinical information from the SEER database and our own center-based data to investigate a novel risk score system for prognostic evaluation in AEG patients. A prognostic risk score signature consisting of six clinical factors (age, grade, tumour size, T stage, M stage, and the ratio of metastatic LNs) was constructed based on the LASSO regression model and showed good predictive ability for the overall survival (OS) of AEG patients in the training and validation sets. Moreover, we revealed that the benefits of chemotherapy and radiotherapy were limited to stage III/IV AEG patients from the high-risk group. After validation in a cohort from our center, this risk score system was also proven to be effective in the prognostic evaluation of AEG. Therefore, our results may provide new insights into the prognostic evaluation and an accurate prognostic biomarker for AEG.

## Materials and methods

### Data source and patients

The SEER database of the National Cancer Institute is an authoritative source of information on cancer incidence and survival, containing data on various tumour sites and from sources throughout the United States (https://seer.cancer.gov/). By using SEER ∗ Stat 8.3.8 software, we obtained demographic information, cancer incidence data, treatment descriptions, and survival data collected from the SEER 18 Regs Custom Database (with additional treatment fields), Nov 2018 Sub (1975–2016 varying). The inclusion criteria were as follows: 1) patients with adenocarcinoma located in the oesophagogastric junction (CS Schema V0204 encoded 28 [EsophagusGEJunction]); 2) patients who were diagnosed via positive histology; 3) patients diagnosed after 2010 (because we used the AJCC 7th (2010) edition for this study); 4) the histology coding was in accordance with the International Classification of Diseases for Oncology 3rd edition (ICD-O-3) within the range of 8140–8145, 8210, 8211, 8220, 8221, 8255, 8260–8263, 8310, and 8480, 8481 and 8490; 5) patients with no other primary tumour except for AEG; 6) patients who received surgery and complete pathological information can be achieved; and 7) patients whose survival information was recorded. We excluded patients 1) for whom we lacked information on age, sex, histological grade, tumour size, radiation and chemotherapy status, number of positive regional nodes and number examined, tumour-node-metastases (TNM) status, vital status, and survival time; 2) aged < 18 years old and survival period < 1 month; and 3) with no specific code of CS tumour size, and number of positive regional nodes and number examined. Here, histological grade was involved in well, moderately, poorly differentiated and undifferentiated groups. According to X-tile software (version 3.6.1) [19], tumour size was optimally categorized as ≤1, 1–2, 2–3, 3–4, 4–5, and > 5 cm.

### The incidence trends of AEG in the SEER database

To explore the incidence rates of AEG, we used SEER ∗ Stat (Version 8.3.8) and Joinpoint (version 4.8.0.1) software [20] to analyse trends in the SEER database from 1975 and 2017. Scatter plots and fitting curves were generated to represent the incidence of AEG during the above years.

### Analysis of prognostic-associated clinical features

First, all AEG patients in the SEER database were randomly divided into two groups: 80% comprised the training set (*n* = 1544) and 20% comprised the internal validation set (*n* = 386). To facilitate our subsequent construction of a prognostic model, we converted clinical categorical variables into numerical variables (e.g., stage 1 into number 1 and female into 0). We provide a supplementary table of transcoding in this study (**Supplementary Table** **1**). In the univariate Cox analysis, we considered only a total of eight clinical features: age, sex, grade, tumour size, T stage, M stage, positive LNs, and the ratio of metastatic LNs (positive LNs/examined LNs). Significant prognostic features (*P*-value < 0.05) were identified by the univariate Cox analysis with the survival package in R.

### Construction of a novel prognostic risk score system

By using the glmnet package in R [21], we generated the LASSO Cox regression model via the classical and modified method, a kind of compression estimation. LASSO compresses some regression coefficients by constructing a penalty function, that is, the sum of the absolute values of the mandatory coefficients is less than a fixed value, and some regression coefficients are set to zero [22]. We used the seven prognostic-associated clinical features described above in the LASSO analysis. After 1000 resamples of the data points of the training set, a set of 1000 matrices was generated. Finally, a list of significant features was selected by the above steps.

Then, the patients in the training set were stratified into low- and high-risk groups according to the best cut-off value of the risk score using X-tile [19]. This software was developed at Yale University and is a graphical method. It shows the presence of a large number of tumour subcohorts and the robustness of the relationship between biomarkers and survival outcomes by constructing a two-dimensional projection of each possible subcohort. Patient survival was analysed using the Kaplan-Meier method and the log-rank test based on the survival package in R. The specificity and sensitivity of the risk score in predicting 1-, 3- and 5-year survival were determined by receiver operating characteristic (ROC) curves using the survivalROC package in R, and the areas under the curve (AUCs) were calculated. The AUC is a summary measure of the ROC curve, reflecting the ability of a test to differentiate results at all possible levels of positivity. We considered that if the AUC was greater than 0.7, the model had good prognostic value.

### Associations of the risk score system and clinicopathological factors

To identify the associations of the risk score according to different clinicopathological factors, scatter plots were drawn to visualise the distribution of risk scores. We predicted 1-, 3- and 5-year survival with the ROC curves and compared these results to those using the traditional TNM staging system.

### External validation cohort from our center

To further validate our novel risk score system, we retrospectively collected data from the Electronic Medical Record System of the Second Affiliated Hospital of Zhejiang University School of Medicine from January 2011 to December 2018. The eligibility criteria were the same as the inclusion criteria for the SEER database. The retrospectively collected data of these patients included demographic parameters, histopathologic tumour characteristics, operation methods, and survival times. Finally, the validation cohort from our center included 174 AEG patients who were recruited according to the inclusion and exclusion criteria. The last follow-up was March 2019. All patients provided written informed consent, and the study was approved by the human research ethics committee of the hospital. Here, we used the AJCC 7th (2010) edition for TNM staging due to its comparative consistency.

### Statistical analysis

All statistical analyses were performed using R language (version 3.6.1). When comparing two independent non-parametric samples, we used the Wilcoxon test, and when comparing multiple independent samples, we used the Kruskal-Wallis test. Univariate Cox regression analysis was used to select prognostic clinical factors. Kaplan-Meier survival plots and log-rank tests were used to compare differences between the high- and low-risk groups. A *P*-value < 0.05 was considered statistically significant.

## Results

### Overall AEG patients’ clinical demographic characteristics

**Fig. 1:**
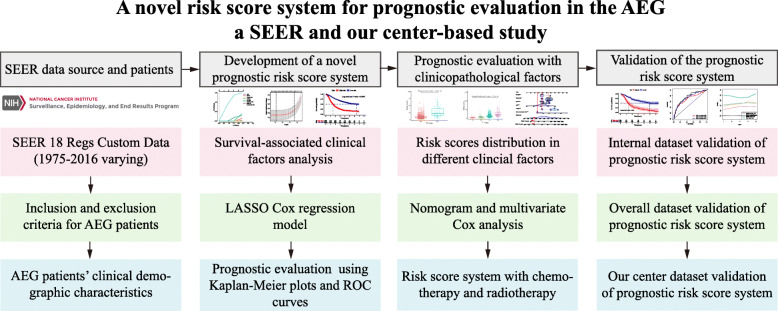
Flow chart of the development of our novel prognostic risk score system for AEG

In this study, we developed a novel risk score system for prognostic evaluation in AEG patients (Fig. 1). The age-adjusted incidence of AEG increased steadily from 1975 to 2016 in the SEER database. This phenomenon occurred in both sex groups, but a slightly higher incidence of AEG was observed in females than in males (**Supplementary Fig. 1A**). This phenomenon also occurred among other clinical factor groups, such as race, grade, and tumour site (**Supplementary Fig. 1B-D**).

Based on the above strict screening conditions, we extracted the clinicopathological variables, including age, sex, histological grade, tumour size, pathological T stage, N stage, M stage, number of positive LNs, ratio of metastatic LNs (positive LNs/examined LNs), survival time and status, of 1930 AEG patients from 2010 to 2016. In the training (*n* = 1544) and internal validation (*n* = 386) sets, the differences between groups were not statistically significant, suggesting that the two groups of patients were random in grouping. The OS time was 24 months in all AEG patients. In addition, 994 (51.5%) patients were alive, and 936 (48.5%) died. The median age in the whole cohort was 63 years, constituting 356 (18.4%) females and 1574 (81.6%) males. Most patients had a poorly differentiated status (54.5%), followed by moderately differentiated (37.7%), well differentiated (6.2%) and undifferentiated (1.6%) statuses. Regarding the clinical TNM stage, 51.1% of patients were at stage III, 24.5% were at stage II, 19.3% were at stage III, and 5.1% were at stage IV. The T stage ranged from T1 to T4 (*n* = 389, 270, 1136, and 135), the N stage ranged from N0 to N3 (*n* = 716, 632, 344, and 238), and the M stage ranged from M0 and M1 (*n* = 1831 and 99). Regarding the chemotherapy status, 1388 (71.9%) patients received chemotherapy. Moreover, approximately half of AEG patients (55.5%) received radiation. The details of the baseline characteristics of the two cohorts are shown in Table 1.

**Table 1:** Clinical characteristics of AEG patients in the training and internal validation set

Clinical features	Overall	Training set	Internal validation set	Statistical test ***p***-value
**Sample size**	1930	1544	386	
**Survival time (months, median [IQR])**	24.00 [13.00, 43.00]	24.00 [13.00, 43.00]	23.00 [13.00, 42.00]	0.826
**Survival status (%)**				0.473
Alive	994 (51.5)	802 (51.9)	192 (49.7)	
Dead	936 (48.5)	742 (48.1)	194 (50.3)	
**Age (years, median [IQR])**	63.00 [55.00, 70.00]	64.00 [56.00, 71.00]	63.00 [55.00, 70.00]	0.396
**Sex (%)**				0.155
Female	356 (18.4)	295 (19.1)	61 (15.8)	
Male	1574 (81.6)	1249 (80.9)	325 (84.2)	
**Histologic grade (%)**				0.952
Well differentiated	120 (6.2)	95 (6.2)	25 (6.5)	
Moderately differentiated	728 (37.7)	582 (37.7)	146 (37.8)	
Poorly differentiated	1051 (54.5)	841 (54.5)	210 (54.4)	
Undifferentiated	31 (1.6)	26 (1.7)	5 (1.3)	
**Tumor size (%)**				0.203
≤1 cm	155 (8.0)	120 (7.8)	35 (9.1)	
1-2 cm	294 (15.2)	235 (15.2)	59 (15.3)	
2-3 cm	356 (18.4)	278 (18.0)	78 (20.2)	
3-4 cm	337 (17.5)	259 (16.8)	78 (20.2)	
4-5 cm	276 (14.3)	228 (14.8)	48 (12.4)	
> 5 cm	512 (26.5)	424 (27.5)	88 (22.8)	
**pStage (%)**				0.244
I	373 (19.3)	286 (18.5)	87 (22.5)	
II	472 (24.5)	378 (24.5)	94 (24.4)	
III	986 (51.1)	803 (52.0)	183 (47.4)	
IV	99 (5.1)	77 (5.0)	22 (5.7)	
**pT stage (%)**				0.209
T1	389 (20.2)	297 (19.2)	92 (23.8)	
T2	270 (14.0)	215 (13.9)	55 (14.2)	
T3	1136 (58.9)	924 (59.8)	212 (54.9)	
T4	135 (7.0)	108 (7.0)	27 (7.0)	
**pN stage (%)**				0.5
N0	716 (37.1)	562 (36.4)	154 (39.9)	
N1	632 (32.7)	510 (33.0)	122 (31.6)	
N2	344 (17.8)	283 (18.3)	61 (15.8)	
N3	238 (12.3)	189 (12.2)	49 (12.7)	
**pM stage (%)**				0.661
M0	1831 (94.9)	1467 (95.0)	364 (94.3)	
M1	99 (5.1)	77 (5.0)	22 (5.7)	
**Radiation status (%)**				0.267
No/Unknown	859 (44.5)	677 (43.8)	182 (47.2)	
Yes	1071 (55.5)	867 (56.2)	204 (52.8)	
**Chemotherapy status (%)**				0.201
No/Unknown	542 (28.1)	423 (27.4)	119 (30.8)	
Yes	1388 (71.9)	1121 (72.6)	267 (69.2)	
**Positive LNs number (median [IQR])**	1.00 [0.00, 3.00]	1.00 [0.00, 3.00]	0.00 [0.00, 3.00]	0.669
**Examined LNs number (median [IQR])**	16.00 [10.00, 23.00]	16.00 [10.00, 23.00]	16.00 [10.00, 23.00]	0.533
**Ratio of metastasis LNs (median [IQR])**	0.03 [0.00, 0.20]	0.03 [0.00, 0.22]	0.00 [0.00, 0.18]	0.369

* The statistical differences between two groups were tested by χ2 or Fisher exact tests, if appropriate. IQR: interquartile range. LNs: lymph nodes

### Development of a novel prognostic risk score system with the LASSO model

**Fig. 2:**
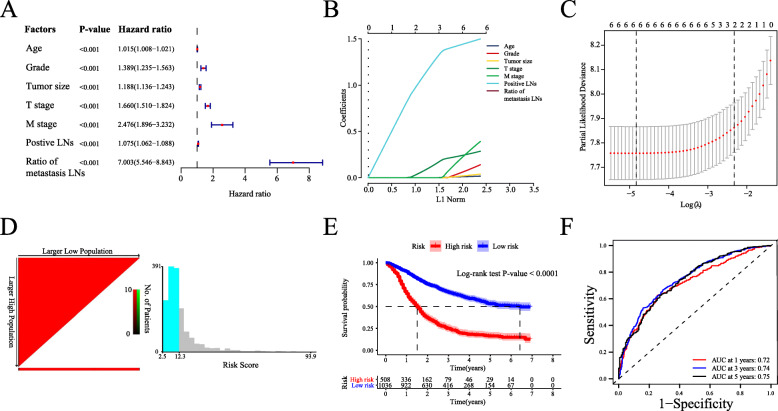
Development of a novel prognostic risk score system. (**A**) Forest plot of prognostic features by using univariate Cox regression analysis. The hazard ratio and its 95% confidence interval are displayed. (**B**) The lambda plot in the LASSO model. The upper coordinate corresponding to the lowest point of the curve is the number of variables ultimately included in the model. (**C**) The cvfit plot in the LASSO model. According to the number of variables included, a vertical line is drawn at the position of the corresponding penalty value, and each curve represents a variable. The vertical coordinate of the variable is the regression coefficient of the variable. (**D**) Estimation of the best cut-off value for the risk score determined with X-tile software. (**E**) Kaplan-Meier plots showing worse survival in the high-risk group than in the low-risk group in the training set. (**F**) The 1-, 3-, and 5-year ROC curves showing the prognostic evaluation performance of our risk score system

In our study, all AEG patients were randomly divided into two groups. In the training set (*n* = 1544), by using univariate Cox regression analysis, we first investigated the prognostic factors for the survival of patients. A total of seven clinical features, namely, age, grade, tumour size, T stage, M stage, positive LNs, and the ratio of metastatic LNs, were identified as prognostic factors according to the univariate analysis (Fig. 2A). We found that all hazard ratios (HRs) of the above prognostic features were greater than 1, suggesting that these factors are clinical risk features for AEG patients. Next, based on the LASSO Cox regression model, we established a risk score system comprising six clinical features (age, grade, tumour size, T stage, M stage, and the ratio of metastatic LNs) for prognostic evaluation in AEG patients. This method allowed us to compute each patient’s risk score by combining the clinical features with the risk coefficient. Here, we chose and shrunk the features with high correlation to prevent overfitting (Fig. 2B and C). The risk scores were then calculated for each patient in the training group, and the patients were assigned to the high-risk or low-risk group based on the most appropriate risk score (12.29 according to X-tile software) (Fig. 2D). As shown in Fig. 2E, patients with high risk scores had significantly worse survival outcomes than those with low risk scores (log-rank test, *P*-value < 0.0001). Furthermore, the AUCs of the risk score for 1-, 3-year and 5-year OS were 0.72, 0.74 and 0.75, respectively (Fig. 2F). The above results proved that our risk score system can be a good predictor of AEG patient survival.

### Prognostic value of the risk score system according to clinicopathological factors

**Fig. 3:**
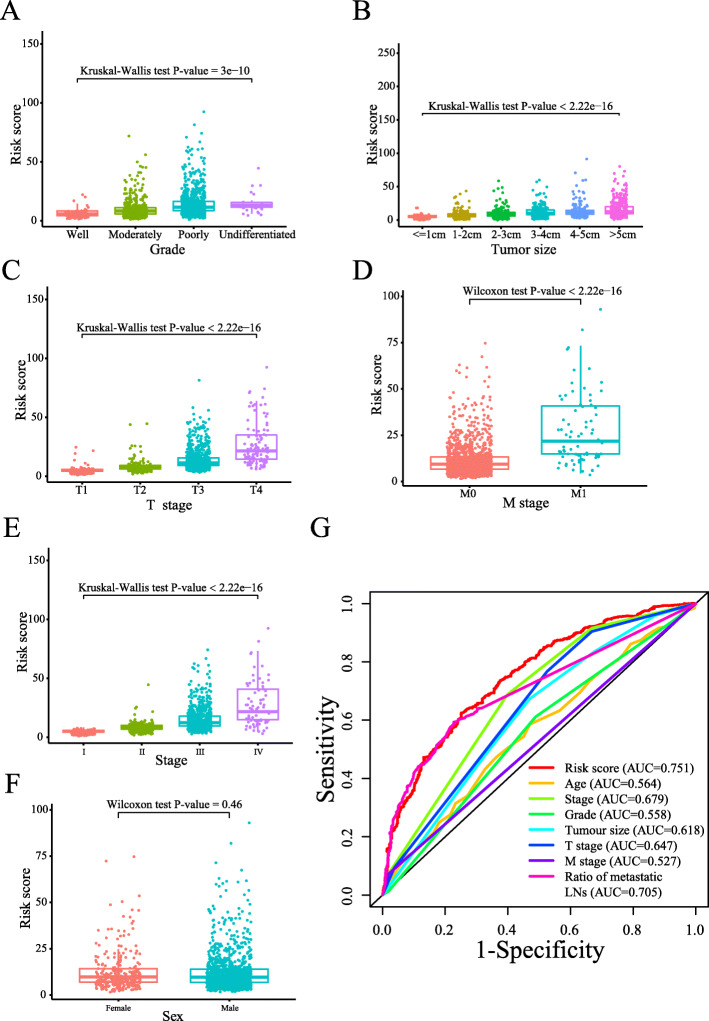
Prognostic value of the risk score system according to clinicopathological factors. The distribution of risk scores according to different clinical features, including grade (**A**), tumour size (**B**), T stage (**C**), M stage (**D**), stage (**E**), and sex (**F**). (**G**) The AUC of our risk score system and traditional TNM staging system as well as other clinical features

To explore the relationships between our risk score system and clinicopathological factors, we examined the risk score differences according to different clinicopathological features. The distribution of risk scores was significantly different according to tumour grade, tumour size, T stage, M stage and TNM stage (*P*-value < 0.0001, Fig. 3A-E). However, there was no significant difference in the distribution of risk scores between female and male AEG patients (Fig. 3F).

To evaluate the prognostic value of our risk score system, ROC analysis was performed based on TNM stage. In Fig. 3G, our risk score system was better than the traditional TNM staging system as well as other clinical features for prognostic evaluation. Combined with other clinical factors, including sex and the number of positive LNs, our risk score system can be considered an independent prognostic factor (**Supplementary Fig. 2B****)**.

### Prognostic value of the risk score system according to chemotherapy and radiotherapy

**Fig. 4:**
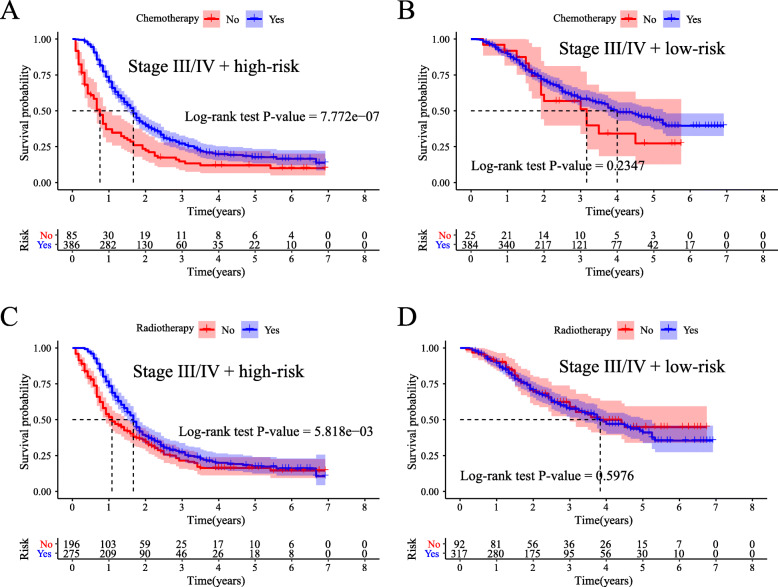
Prognostic value of the risk score system according to chemotherapy and radiotherapy. (**A**) Kaplan-Meier plots of stage III/IV patients in the high-risk group who did or did not receive chemotherapy. (**B**) Kaplan-Meier plots of stage III/IV patients in the low-risk group who did or did not receive chemotherapy. (**C**) Kaplan-Meier plots of stage III/IV patients in the high-risk group who did or did not receive radiotherapy. (**D**) Kaplan-Meier plots of stage III/IV patients in the low-risk group who did or did not receive radiotherapy

In the SEER 18 Regs Custom Database, we can also obtain information on additional treatment fields, such as chemotherapy and radiotherapy. Thus, to evaluate the prognostic value of the risk score system, Kaplan–Meier and stratification analyses were performed according to TNM stage and the receipt of chemotherapy and radiotherapy. After stratification by TNM stage, our risk score system was significantly correlated with AEG prognosis. Patients in the high-risk group with stage III or IV disease had a better prognosis when they received chemotherapy than when they did not (log-rank test, *P*-value < 0.0001, Fig. 4A), whereas patients in the low-risk group had no significant difference in prognosis with or without chemotherapy (log-rank test, P-value > 0.05, Fig. 4B). Similar results were also observed with radiation. AEG patients in the high-risk group with stage III or IV disease had a better prognosis when they received radiotherapy (log-rank test, *P*-value < 0.0001, Fig. 4C). Patients in the low-risk group had no significant difference in prognosis with or without radiotherapy (log-rank test, P-value > 0.05, Fig. 4D). Therefore, our findings revealed that the benefits of chemotherapy and radiotherapy were limited to stage III/IV AEG patients from the high-risk group.

### Internal and external validation of the prognostic risk score system

**Fig. 5:**
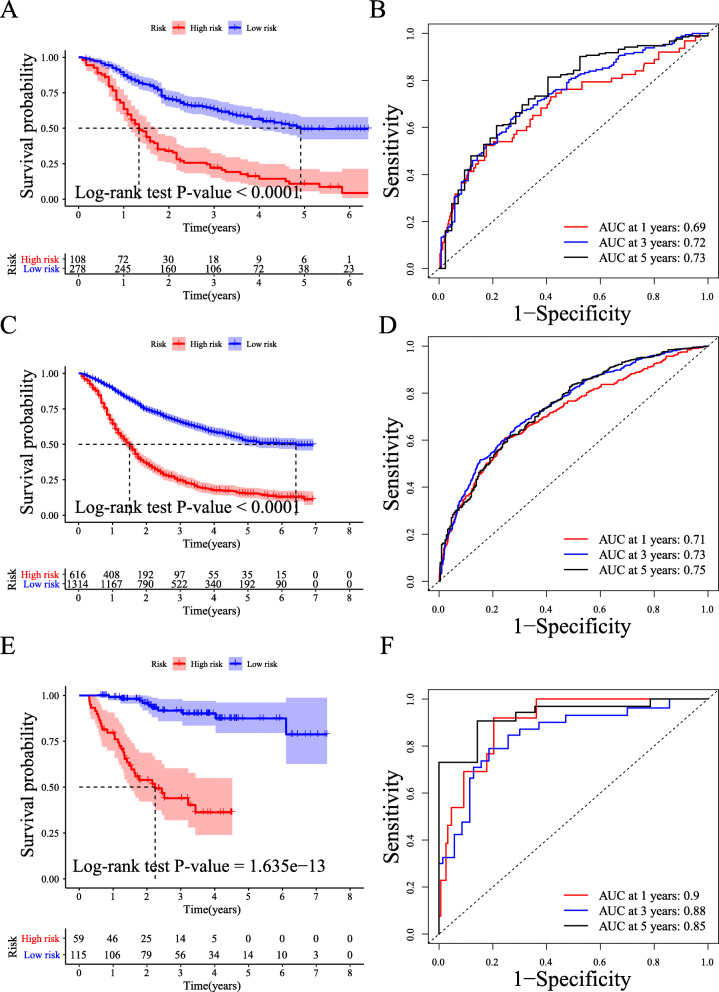
Internal and external validation of the prognostic risk score system. (**A**) Kaplan-Meier plots of the high- and low-risk groups in the internal validation set (*n* = 386). (**B**) The 1-, 3-, and 5-year ROC curves in the internal validation set. (**C**) Kaplan-Meier plots of the high- and low-risk groups in the whole set (*n* = 1930). (**D**) The 1-, 3-, and 5-year ROC curves in the whole set. (**E**) Kaplan-Meier plots of the high- and low-risk groups in the validation set from our center (*n* = 174). (**F**) Kaplan-Meier plots of the high- and low-risk groups in the validation set from our center

To validate the risk score system, its prognostic accuracy was further assessed in the internal and external validation sets. In the internal validation set (*n* = 386), based on the same risk score cut-off, the survival outcome was significantly longer for patients in the low-risk group (log-rank test, *P*-value < 0.0001, Fig. 5A). Then, we drew ROC curves to evaluate the prediction accuracy of our model, with 1-, 3-, and 5-year AUC values of 0.69, 0.72, and 0.73, respectively (Fig. 5B). Moreover, we determined the prediction power of our risk score system in the whole SEER patient dataset (*n* = 1930). The prognostic accuracy of our risk score system was also validated (log-rank test, *P*-value < 0.0001, Fig. 5C), with respective AUCs of 0.73 and 0.75 for 3-year and 5-year survival outcomes (Fig. 5D).

To further validate our novel risk score system, we retrospectively analysed a total of 174 AEG patients from our center from January 2011 to December 2018 (Table 2). According to the same inclusion and exclusion criteria, we obtained similar results. First, we observed different survival outcomes between the high- and low-risk groups based on the same risk score cut-off (log-rank test, *P*-value < 0.0001, Fig. 5E). The AUC values at 1 year, 3 years, and 5 years were 0.9, 0.88, and 0.85, respectively (Fig. 5F). Interestingly, the power of evaluation in our cohort was much better than that in the SEER cohort (**Supplementary Fig. 3A**). Among the 174 AEG patients in our center, the number of recurrence or metastasis patients was 55 (31.6%). According to the risk score, the 3-year recurrence-free survival (RFS) of patients in the low-risk group is 83.5, and 34.2% in the high-risk group. Moreover, we performed a Kaplan–Meier analysis to observe the difference of RFS between two risk groups. As shown in **Supplementary Fig. 3B**, patients with high-risk scores had significantly worse RFS outcomes than those with low-risk scores (log-rank test, P-value < 0.0001). Thus, our risk score system can not only predict the patient’s OS, but also predict the patient’s RFS.

**Table 2:** Clinical characteristics of AEG patients in the SEER and our center set

Clinical features	SEER set	Our center set
**Sample size**	1930	174
**Survival time (months, median [IQR])**	24.00 [13.00, 43.00]	26.67 [16.93, 45.85]
**Survival status (%)**		
Alive	994 (51.5)	132 (75.9)
Dead	936 (48.5)	42 (24.1)
**Age (years, median [IQR])**	63.00 [55.00, 70.00]	65.50 [60.00, 71.00]
**Sex (%)**		
Female	356 (18.4)	34 (19.5)
Male	1574 (81.6)	140 (80.5)
**Histologic grade (%)**		
Well differentiated	120 (6.2)	12 (6.9)
Moderately differentiated	728 (37.7)	68 (39.1)
Poorly differentiated	1051 (54.5)	92 (52.9)
Undifferentiated	31 (1.6)	2 (1.1)
**Tumor size (%)**		
≤1 cm	155 (8.0)	7 (4.0)
1-2 cm	294 (15.2)	23 (13.2)
2-3 cm	356 (18.4)	35 (20.1)
3-4 cm	337 (17.5)	46 (26.4)
4-5 cm	276 (14.3)	23 (13.2)
> 5 cm	512 (26.5)	40 (23.0)
**pStage (%)**		
I	373 (19.3)	43 (24.7)
II	472 (24.5)	63 (36.2)
III	986 (51.1)	63 (36.2)
IV	99 (5.1)	5 (2.9)
**pT stage (%)**		
T1	389 (20.2)	25 (14.4)
T2	270 (14.0)	31 (17.8)
T3	1136 (58.9)	79 (45.4)
T4	135 (7.0)	39 (22.4)
**pN stage (%)**		
N0	716 (37.1)	70 (40.2)
N1	632 (32.7)	35 (20.1)
N2	344 (17.8)	34 (19.5)
N3	238 (12.3)	35 (20.1)
**pM stage (%)**		
M0	1831 (94.9)	169 (97.1)
M1	99 (5.1)	5 (2.9)
**Chemotherapy status (%)**		
No/Unknown	542 (28.1)	40 (23.0)
Yes	1388 (71.9)	134 (77.0)
**Positive LNs number (median [IQR])**	1.00 [0.00, 3.00]	1.00 [0.00, 5.00]
**Examined LNs number (median [IQR])**	16.00 [10.00, 23.00]	30.00 [22.00, 38.00]
**Ratio of metastasis LNs (median [IQR])**	0.03 [0.00, 0.20]	0.05 [0.00, 0.19]

## Discussion

In this study, we identified a novel risk score system for prognostic evaluation in AEG patients based on a large population from the SEER database and a patient cohort from our center We showed that this risk score system, consisted of six clinical features (age, grade, tumour size, T stage, M stage, and the ratio of metastatic lymph nodes), can be a good predictor of AEG patient survival based on the training and validation sets and the set from our center.

In the present study, we first obtained a total of 1930 AEG patients from the SEER database: 1544 and 386 patients as the training and internal validation sets, respectively. Because the sample size and number of samples in the database are very large, our results are reliable. We examined not only AEG but also other human cancers using data from the SEER database [15, 17, 18, 23, 24]. We compared the number of patients with different types of cancer described in the SEER database over the last two years (**Supplementary Table** **2**). From the results, we observed that certain types of cancer or specific types of one common cancer had a relatively fewer number of samples than the more common cancers. Nevertheless, the sample size was still large enough to yield reliable results.

Compared with other similar studies on cancers, most studies have used nomograms to predict OS for patients with cancer. In these studies, univariate and multivariate Cox or logistic regression analyses were usually performed to build one prognostic risk model for patients. However, in our study, we selected the LASSO model to build a risk score system because it has several advantages. LASSO can reduce the effect of collinearity, thereby reducing model variance because of a serious collinearity problem among multiple variables. If a set of variables is highly correlated, this method will select only one variable and shrink the others to zero. Thus, it can aid in feature selection [25]. Regression regularization methods (including the LASSO method) work well in cases of high dimensionality and multicollinearity among the variables in a dataset [26, 27]. LASSO models perform variable selection and regularization to improve predictive accuracy and interpretability [28].

Adjuvant chemotherapy based on a fluorouracil regimen was associated with a lower risk of death from gastric cancer than surgery alone [29]. For elderly patients with locally advanced adenocarcinoma of the stomach and the oesophagogastric junction who are considered candidates for chemotherapy, perioperative treatment seems feasible and effective [30]. In one Japanese study [31], preoperative chemotherapy was shown to be potentially beneficial for Japanese patients with Siewert type II adenocarcinoma. In our study, we found an interesting phenomenon. Regardless of whether it is high-risk or stage III/IV patients, the prognosis of patients receiving chemotherapy and radiotherapy is better than patients who do not receive chemotherapy and radiotherapy (**Supplementary Fig. 4**). Meanwhile, we found that, in stage III/IV AEG patients, the benefits of chemotherapy and radiotherapy were limited to the high-risk group. This means that not all patients will benefit from chemotherapy, not even patients with advanced AEG. Thus, our novel risk score system will allow us to better distinguish which patients with advanced AEG will benefit from chemotherapy (high-risk) and which will not (low-risk). However, given the retrospective nature of our study, the lack of benefit of adjuvant chemotherapy and radiotherapy in stage III/IV but low-risk patients should be interpreted with caution. The major cause of this difference may be selection bias of clinical factors. For example, we found patients who did not receive chemotherapy tend to have older age compared with patients who received chemotherapy. Thus, we will make efforts to prove above results in further study, especially avoiding selection bias.

The greatest advantage of our risk score system is the integration of common clinical variables, and the ability of our system to assess prognosis is far superior to other pathologic factors. A single factor is not sufficient to predict a patient’s prognosis and survival. Also, in our risk score system, we introduced the clinical factor “the ratio of metastatic LNs” instead of traditional N stage. TNM is the main tool for judging the prognosis of gastric cancer, but the number of metastatic LNs may be affected by surgical, pathological, tumor or host factors. Some authors have also shown that the lymph node ratio may be better than TNM staging [32, 33]. Interestingly, we found two similar studies in a literature search from the PubMed database. Zhou et al. [34] used the AEG patients’ information from 1988 to 2011 to construct one nomogram that provided significantly improved discrimination than the traditional AJCC TNM classification. Also, based on data between 2004 and 2010, Wang et al. established a competing risk model for predicting survival of AEG patients [35]. In contrast to the above two studies, our study is innovative as follows (**Supplementary Table** **3**). First, we selected the latest patient data (based on the 7th edition of the AJCC TNM staging system), which most closely resemble those of the 8th edition of the AJCC TNM staging system. Second, the method used in this study (LASSO model) was different from that used in the above two studies (multivariate Cox proportional hazards regression model). The LASSO method can improve predictive accuracy and interpretability. Third, we considered the ratio of metastatic LNs, not N stage or the number of LNs examined. Most importantly, we explored the prognostic value of the risk score system according to chemotherapy and radiotherapy. In addition, in the above two studies, only a nomogram was developed; however, we generated a risk score system to predict the survival outcomes of AEG patients. Therefore, our study has more advantages over the above two studies.

Our work also has some limitations. First, we need to consider other molecular-level indicators, such as genes, proteins and other molecules, in our risk score system to make the predictions of survival outcomes of AEG patients more effective. Second, due to limitations of the SEER database, we were unable to make a full comparison to the latest AJCC 8th classification. Third, no such specific information in the SEER database such as surgical procedure, the range of lymphadenectomy, and the curability of the cases, we were unable to take above important factors into account in our risk score system. Last, our risk score system do not work in preoperative situation. Whether our risk score system can be used to predict the risks of preoperative patients is worthy of further study. Thus, we will gradually improve the above work in follow-up research. In brief, we developed and validated a novel risk score system for prognostic evaluation in AEG patients. Our results may provide new insights into the prognostic evaluation of AEG.

## Conclusion

We developed and validated a novel risk score system for prognostic evaluation in AEG patients. Our results may provide new insights into the prognostic evaluation of AEG.

## Supplementary Information

**Additional file 1 Supplementary Table 1.** Transcoding of each clinical feature in this study.

**Additional file 2 Supplementary Table 2.** Sample sizes of patients with different cancers based on the SEER database over the last two years.

**Additional file 3 Supplementary Table 3.** The comparison of our study and two previous studies on AEG patients.

**Additional file 4 Supplementary Fig. 1.** Annual age-adjusted incidence of AEG. The incidence of AEG by sex (**A**), race (**B**), grade (**C**), and tumour site (**D**).

**Additional file 5 Supplementary Fig. 2.** Prognostic value of the risk score system according to clinicopathological factors. Forest plot of prognostic features by using multivariate Cox regression analysis.

**Additional file 6 Supplementary Fig. 3.** The analysis of AUC comparison and RFS in our center. (**A**) Comparison of AUC values in the training set, internal set, whole set and validation set from our center. (**B**) Kaplan-Meier plots of RFS between high- and low-risk groups in our center (*n* = 174).

**Additional file 7 Supplementary Fig. 4.** Prognostic value of the risk score system and Stage III/IV according to chemotherapy and radiotherapy. (**A**) Kaplan-Meier plots of patients in the high-risk group who did or did not receive chemotherapy. (**B**) Kaplan-Meier plots of Stage III/IV patients who did or did not receive chemotherapy. (**C**) Kaplan-Meier plots of patients in the high-risk group who did or did not receive radiotherapy. (**D**) Kaplan-Meier plots of Stage III/IV patients who did or did not receive radiotherapy.

## Data Availability

All data generated or analyzed during this study are included in this published article and its supplementary information files. The datasets generated and analysed during the current study are available in The Surveillance, Epidemiology, and End Results (SEER) database (https://seer.cancer.gov/).

## References

[1] Sung H, Ferlay J, Siegel RL, Laversanne M, Soerjomataram I, Jemal A (2021). Global cancer statistics 2020: GLOBOCAN estimates of incidence and mortality worldwide for 36 cancers in 185 countries. CA Cancer J Clin..

[2] Hasegawa S, Yoshikawa T (2010). Adenocarcinoma of the esophagogastric junction: incidence, characteristics, and treatment strategies. Gastric Cancer.

[3] Hatta W, Tong D, Lee YY, Ichihara S, Uedo N, Gotoda T (2017). Different time trend and management of esophagogastric junction adenocarcinoma in three Asian countries. Dig Endosc.

[4] Liu K, Yang K, Zhang W, Chen X, Chen X, Zhang B, Chen Z, Chen J, Zhao Y, Zhou Z, Chen L, Hu J (2016). Changes of Esophagogastric junctional adenocarcinoma and gastroesophageal reflux disease among surgical patients during 1988-2012: a single-institution, high-volume experience in China. Ann Surg.

[5] Carr JS, Zafar SF, Saba N, Khuri FR, El-Rayes BF (2013). Risk factors for rising incidence of esophageal and gastric cardia adenocarcinoma. J Gastrointest Cancer.

[6] Imamura Y, Watanabe M, Oki E, Morita M, Baba H (2021). Esophagogastric junction adenocarcinoma shares characteristics with gastric adenocarcinoma: literature review and retrospective multicenter cohort study. Ann Gastroenterol Surg.

[7] Rice TW, Gress DM, Patil DT, Hofstetter WL, Kelsen DP, Blackstone EH (2017). Cancer of the esophagus and esophagogastric junction-major changes in the American joint committee on Cancer eighth edition cancer staging manual. CA Cancer J Clin.

[8] Suh YS, Lee KG, Oh SY, Kong SH, Lee HJ, Kim WH, Yang HK (2017). Recurrence pattern and lymph node metastasis of adenocarcinoma at the Esophagogastric junction. Ann Surg Oncol.

[9] Chen L, Guo X, Chen S, Ren Y, Sun T, Yang F, Zheng C (2021). Comparison of the efficacy of pre-surgery and post-surgery radiotherapy in the treatment of hepatocellular carcinoma: a population-based study. Am J Transl Res.

[10] Poulson MR, Blanco BA, Geary AD, Kenzik KM, McAneny DB, Tseng JF, Sachs TE (2021). The role of racial segregation in treatment and outcomes among patients with hepatocellular carcinoma. HPB (Oxford).

[11] Li T, Chen S, Zhang Z, Lin L, Wu Q, Li J, Lin Q (2021). Chemotherapy plus radiotherapy versus radiotherapy in patients with small cell carcinoma of the esophagus: a SEER database analysis. Cancer Control.

[12] Torrecillas V, Shepherd HM, Francis S, Buchmann LO, Monroe MM, Lloyd S, Cannon D, Hitchcock YJ, Weis JR, Hunt JP, Houlton JJ, Cannon RB (2018). Adjuvant radiation for T1-2N1 oral cavity cancer survival outcomes and utilization treatment trends: analysis of the SEER database. Oral Oncol.

[13] Zhong Q, Shi Y (2020). Development and validation of a novel risk stratification model for Cancer-specific survival in diffuse large B-cell lymphoma. Front Oncol.

[14] Dashti NK, Cates JMM. Risk assessment of visceral sarcomas: a comparative study of 2698 cases from the SEER database. Ann Surg Oncol. 2021. 10.1245/s10434-020-09576-2. 10.1245/s10434-020-09576-2 33538930

[15] Wang R, Xie G, Shang L, Qi C, Yang L, Huang L, Li D, Yang W (2021). Development and validation of nomograms for epithelial ovarian cancer: a SEER population-based, real-world study. Future Oncol.

[16] Li H, Cai Z, Liu R, Hu J, Chen J, Zu X (2021). Clinicopathological characteristics and survival outcomes for testicular choriocarcinoma: a population-based study. Transl Androl Urol.

[17] Lu YJ, Duan WM (2021). Establishment and validation of a novel predictive model to quantify the risk of bone metastasis in patients with prostate cancer. Transl Androl Urol.

[18] Luo T, Wang Y, Shan X, Bai Y, Huang C, Li G, Wang H (2021). Nomogram based on homogeneous and heterogeneous associated factors for predicting distant metastases in patients with colorectal cancer. World J Surg Oncol.

[19] Camp RL, Dolled-Filhart M, Rimm DL (2004). X-tile: a new bio-informatics tool for biomarker assessment and outcome-based cut-point optimization. Clin Cancer Res.

[20] Kim HJ, Fay MP, Feuer EJ, Midthune DN. Permutation tests for joinpoint regression with applications to cancer rates. Stat Med. 2000;19(3):335–51. 10.1002/(SICI)1097-0258(20000215)19:3<335::AID-SIM336>3.0.CO;2-Z. 10.1002/(sici)1097-0258(20000215)19:3<335::aid-sim336>3.0.co;2-z 10649300

[21] Friedman J, Hastie T, Tibshirani R (2010). Regularization paths for generalized linear models via coordinate descent. J Stat Softw.

[22] Tibshirani R. The lasso method for variable selection in the cox model. Stat Med. 1997;16(4):385–95. 10.1002/(SICI)1097-0258(19970228)16:4<385::AID-SIM380>3.0.CO;2-3. 10.1002/(sici)1097-0258(19970228)16:4<385::aid-sim380>3.0.co;2-3 9044528

[23] Tang J, Jiang S, Gao L, Xi X, Zhao R, Lai X, Zhang B, Jiang Y (2021). Construction and validation of a nomogram based on the log odds of positive lymph nodes to predict the prognosis of medullary thyroid carcinoma after surgery. Ann Surg Oncol.

[24] Zhang M, Lei S, Chen Y, Wu Y, Ye H (2021). The role of lymph node status in cancer-specific survival and decision-making of postoperative radiotherapy in poorly differentiated thyroid cancer: a population-based study. Am J Transl Res.

[25] Li Y, Hong HG, Ahmed SE, Li Y (2019). Weak signals in high-dimension regression: detection, estimation and prediction. Appl Stoch Models Bus Ind.

[26] Klosa J, Simon N, Westermark PO, Liebscher V, Wittenburg D (2020). Seagull: lasso, group lasso and sparse-group lasso regularization for linear regression models via proximal gradient descent. BMC Bioinformatics.

[27] Greenwood CJ, Youssef GJ, Letcher P, Macdonald JA, Hagg LJ, Sanson A, McIntosh J, Hutchinson DM, Toumbourou JW, Fuller-Tyszkiewicz M (2020). A comparison of penalised regression methods for informing the selection of predictive markers. PLoS One.

[28] Pripp AH, Stanisic M (2017). Association between biomarkers and clinical characteristics in chronic subdural hematoma patients assessed with lasso regression. PLoS One.

[29] Paoletti X, Oba K, Burzykowski T, Michiels S, Ohashi Y, Pignon JP, Rougier P, Sakamoto J, Sargent D, Group G (2010). Benefit of adjuvant chemotherapy for resectable gastric cancer: a meta-analysis. JAMA.

[30] Haag GM, Byl A, Jager D, Berger AK (2017). Perioperative chemotherapy in elderly patients with locally advanced adenocarcinoma of the stomach and the Esophagogastric junction: a retrospective cohort analysis of toxicity and efficacy at the National Center for tumor diseases, Heidelberg. Oncology.

[31] Hosoda K, Yamashita K, Katada N, Moriya H, Mieno H, Sakuramoto S, Kikuchi S, Watanabe M (2015). Benefit of neoadjuvant chemotherapy for Siewert type II esophagogastric junction adenocarcinoma. Anticancer Res.

[32] Zhu J, Xue Z, Zhang S, Guo X, Zhai L, Shang S, Zhang Y, Lu H (2018). Integrated analysis of the prognostic role of the lymph node ratio in node-positive gastric cancer: a meta-analysis. Int J Surg.

[33] Spolverato G, Ejaz A, Kim Y, Squires MH, Poultsides G, Fields RC, Bloomston M, Weber SM, Votanopoulos K, Acher AW, Jin LX, Hawkins WG, Schmidt C, Kooby DA, Worhunsky D, Saunders N, Cho CS, Levine EA, Maithel SK, Pawlik TM (2015). Prognostic performance of different lymph node staging systems after curative intent resection for gastric adenocarcinoma. Ann Surg.

[34] Zhou Z, Zhang H, Xu Z, Li W, Dang C, Song Y (2015). Nomogram predicted survival of patients with adenocarcinoma of esophagogastric junction. World J Surg Oncol.

[35] Wang T, Wu Y, Zhou H, Wu C, Zhang X, Chen Y, Zhao D (2021). Development and validation of a novel competing risk model for predicting survival of esophagogastric junction adenocarcinoma: a SEER population-based study and external validation. BMC Gastroenterol.

