# Supplementary figures and images for: A novel risk score system for prognostic evaluation in adenocarcinoma of the oesophagogastric junction: a large population study from the SEER database and our center
Source: BMC Cancer. 2021 Jul 13;21:806. doi: 10.1186/s12885-021-08558-1 (PMC8278582; doi:10.1186/s12885-021-08558-1)

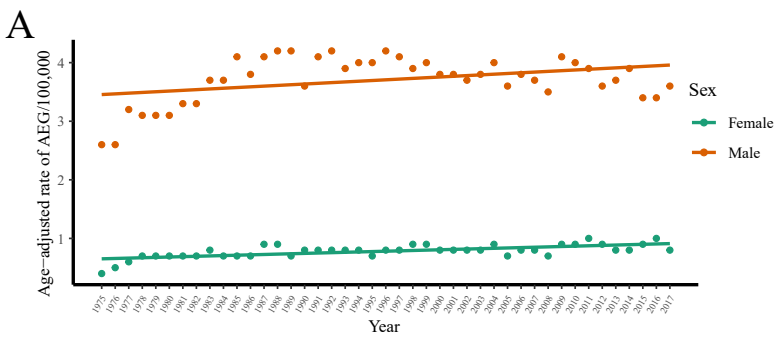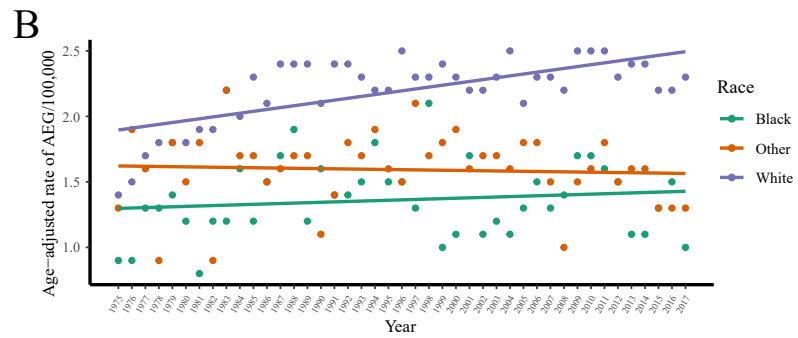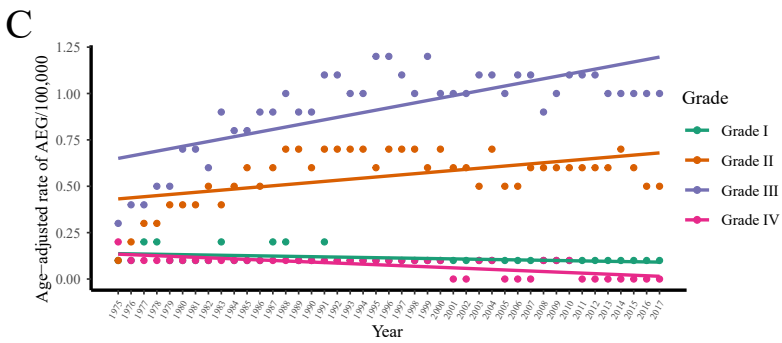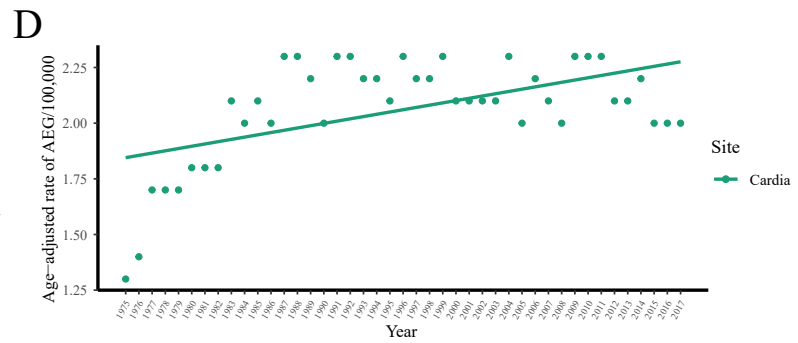

Supplement: Supplementary file 4 — Additional file 4 Supplementary Fig. 1. Annual age-adjusted incidence of AEG. The incidence of AEG by sex (A), race (B), grade (C), and tumour site (D). [file 12885_2021_8558_MOESM4_ESM.pdf]

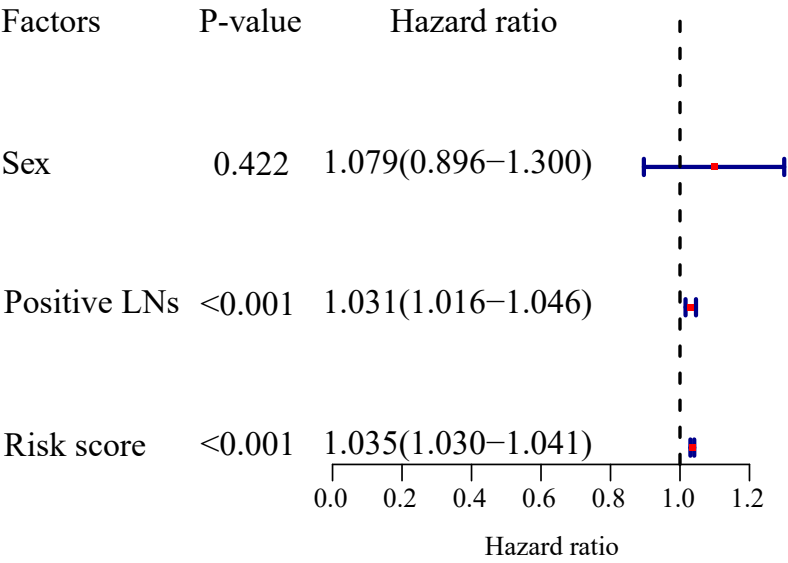

Supplement: Supplementary file 5 — Additional file 5 Supplementary Fig. 2. Prognostic value of the risk score system according to clinicopathological factors. Forest plot of prognostic features by using multivariate Cox regression analysis. [file 12885_2021_8558_MOESM5_ESM.pdf]

A

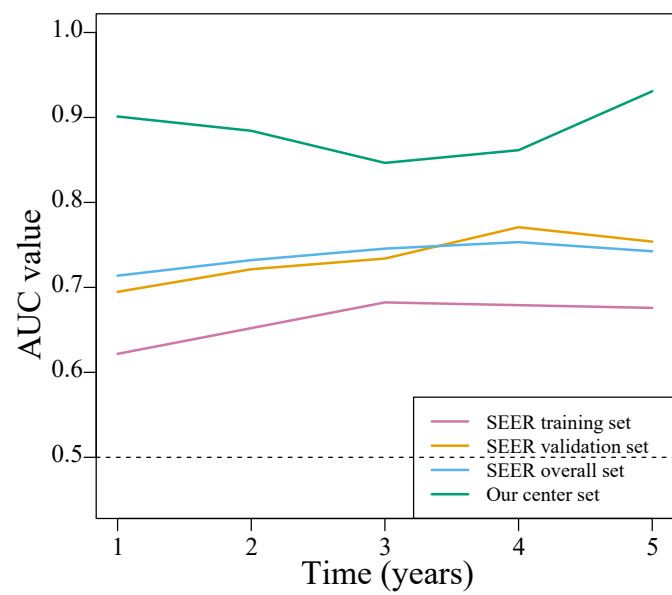

B

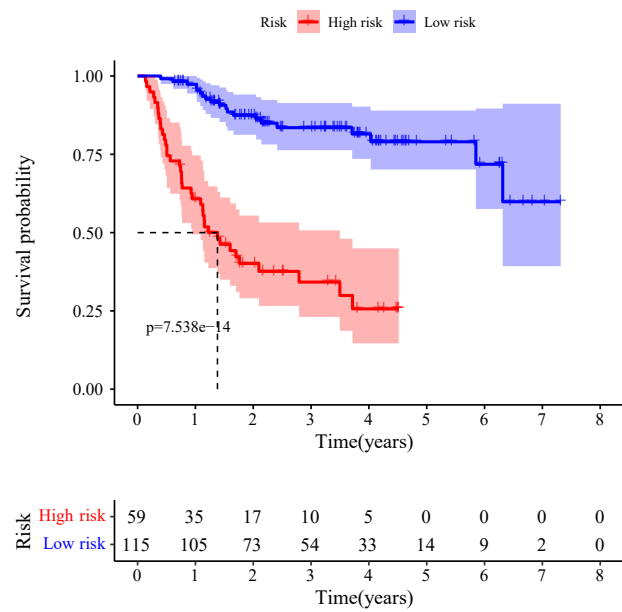

Supplement: Supplementary file 6 — Additional file 6 Supplementary Fig. 3. The analysis of AUC comparison and RFS in our center. (A) Comparison of AUC values in the training set, internal set, whole set and validation set from our center. (B) Kaplan-Meier plots of RFS between high- and low-risk groups in our center (n = 174). [file 12885_2021_8558_MOESM6_ESM.pdf]

A

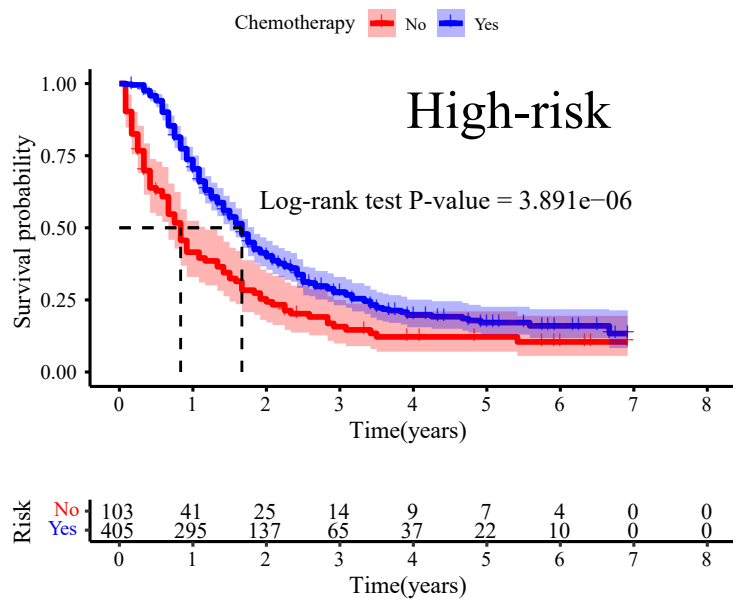

B

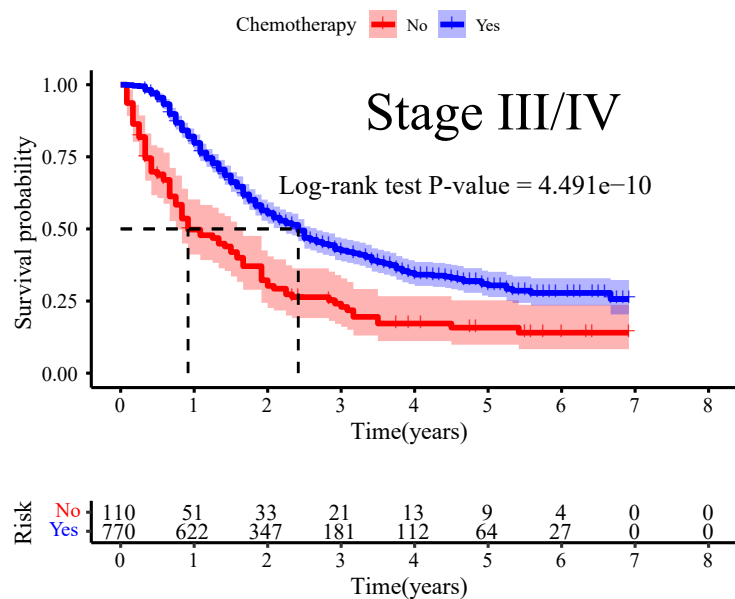

C

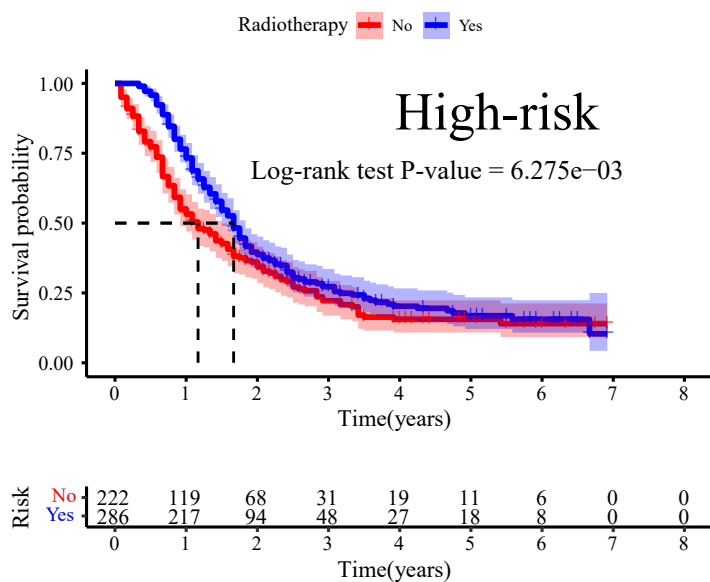

D

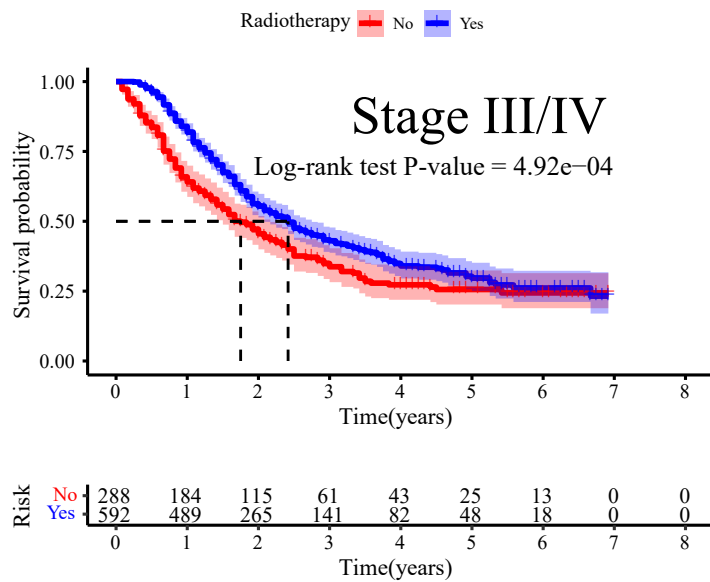

Supplement: Supplementary file 7 — Additional file 7 Supplementary Fig. 4. Prognostic value of the risk score system and Stage III/IV according to chemotherapy and radiotherapy. (A) Kaplan-Meier plots of patients in the high-risk group who did or did not receive chemotherapy. (B) Kaplan-Meier plots of Stage III/IV patients who did or did not receive chemotherapy. (C) Kaplan-Meier plots of patients in the high-risk group who did or did not receive radiotherapy. (D) Kaplan-Meier plots of Stage III/IV patients who did or did not receive radiotherapy. [file 12885_2021_8558_MOESM7_ESM.pdf]
